# Thyroid Carcinoma in Children and Adolescents—Systematic Review of the Literature

**DOI:** 10.4061/2011/845362

**Published:** 2011-09-04

**Authors:** Fernanda Vaisman, Rossana Corbo, Mario Vaisman

**Affiliations:** ^1^Endocrinology Service, Universidade Federal do Rio de Janeiro, Rio de Janiro, RJ, Brazil; ^2^Endocrinology Service, Instituto Nacional do Cancer, Rio de Janeiro, Rio de Janiro, RJ, Brazil

## Abstract

Thyroid cancer in children and adolescents is usually a major concern for physicians, patients, and parents. Controversies regarding the aggressiveness of the clinical presentation and the ideal therapeutic approach remain among the scientific community. The current recommendations and staging systems are based on data generated by studies in adults, and this might lead to overtreating in some cases as well as undertreating in others. Understanding the differences in the biology, clinical course, and outcomes in this population is crucial for therapeutic decisions. This paper evaluates the biology, clinical presentation, recurrences, and overall survival as well as the staging systems in children and adolescents with differentiated thyroid cancer.

## 1. Introduction

Palpable thyroid nodules can be diagnosed in 4 to 7% of the adult population. The high-resolution ultrasounds are able to detect nodules around 19% of the adult population, reaching up to 67% in populations at higher risk such as women and elderly individuals [1]. Considering autopsy series, this prevalence can reach 50%. Although common, only 5% are malignant [2]. 

Thyroid cancer is a rare pathology in childhood and adolescence being responsible for 1.5–3% of all carcinomas in this age group in the USA and Europe [3]. Such as the adults, the differentiated thyroid carcinoma is the most commonly found, especially the papillary carcinoma. In this population, age, family history of thyroid disease and radiation exposure are very important factors as already shown in various series [4–6], especially after the Chernobyl accident, when a substantial increase in the incidence of thyroid carcinoma in children exposed to radiation was documented [7]. 

Staging thyroid carcinoma in children and adolescents is still a controversial issue. To avoid overtreating, a risk classification system, with the highest accuracy as possible, should be used to identify patients who should be treated in a more conservative or more aggressive way. 

The current treatment recommendation is the total thyroidectomy followed by radioiodine therapy, based on good response and high disease-free survival rate for this age group. However, many authors question the aggressiveness of this treatment given the long lifespan of these patients and long-term complications of high doses of radioiodine. 

This revision aims to evaluate the initial therapeutic approach for children and adolescents with DTC regarding surgery, adjuvant therapy, and staging.

## 2. Epidemiology of the Disease

The incidence of clinically palpable thyroid nodules in children is estimated to be around 1–1.5%. However, in teenagers, this prevalence may reach 13% [8]. When compared to adults, children have four times greater risk of malignancy when a thyroid nodule is diagnosed. In the US, around 350 individuals aged less than 20 years receive the diagnosis of thyroid carcinoma annually [9]. In Brazil, the incidence can reach 2% of all pediatric cancers according to the National Cancer Institute database [10].

Besides being a rare disease, the differentiated thyroid carcinoma accounts for about 0.5–3% of all malignancies in the pediatric population [8]. In addition, the thyroid is one of the most common sites of a second primary tumor in children who received external beam radiotherapy to the neck for the treatment of other neoplasms. 

The occurrence of thyroid carcinoma in early childhood is very rare. In the literature, there are isolated cases of differentiated thyroid carcinoma in neonates and infants aged less than 1 year old [11, 12]. 

Furthermore, the incidence of thyroid cancer seems to increase with age. In a series with 235 children and adolescents who followed Maria Skłodowska Memorial Cancer Center and Institute of Oncology for thyroid cancer, 5% were diagnosed under 6 years old, 10% with 7–9 years, increasing substantially after 10 years old. The difference between boys and girls was seen more clearly after 13-14 years old [13]. Also the latest records of SEER cohort (Surveillance, Epidemiology and End Results) from a group of 1753 patients aged less than 20 years confirm the greater incidence in girls (0.89 cases/100,000 for girls versus 0.2 cases/100,000 for boys) [14].

## 3. Risk Factors

In the past 60 years, the incidence of thyroid carcinoma in the pediatric age group presented two distinct peaks. The first occurred around 1950 due to the use of radiation for the treatment of common childhood conditions such as *Tinea capitis*, acne, chronic tonsillitis, and thymus hyperplasia [15, 16]. In these cases, the thyroid carcinoma was diagnosed on average 10–20 years after exposure, but with risk persisting until 40 years later. When the causal relationship between neck irradiation and thyroid carcinoma was established, such practices were abandoned leading to a decreasing incidence in this population [11]. These data led to acceptance of ionizing radiation, a risk factor for the development of thyroid cancer [17]. Similarly, external beam radiotherapy for the treatment of other childhood malignancies would also be associated with an increased incidence of thyroid carcinoma in this population [18–20]. 

A second peak incidence occurred in the mid-1990s in some regions of Eastern Europe on behalf of the nuclear accident that occurred in Chernobyl in 1986 [4–6]. The first cases were diagnosed approximately 4-5 years later, especially in children under 5 years old at the time of exposure [4, 21]. About 75% of these cases were exposed to the radioactive fallout between birth and 14 years of age, with most of the other 25% being from 14 to 17 years old at the time of exposure [21]. The Chernobyl accident confirmed the higher sensitivity of the pediatric population, to the effects of radiation when compared to adults [22]. 

The effects of ionizing radiation on thyroid remain of great interest of the scientific community. The British Childhood Cancer Survivor Study (BCCSS) is a cohort of 17,980 patients who were followed on average for 17.4 years, so far, whose main objective is to determine the occurrence of a second primary tumor. Eighty-eight percent of thyroid carcinomas were found in patients undergoing radiotherapy covering the cervical region. The risk of thyroid carcinoma was higher in patients treated for Hodgkin's disease (RR 3.3—IC: 1.1–10.1) and non-Hodgkin Lymphoma (RR 3.4—IC: 1.1–10.7) [23].

## 4. Presentation in Childhood

Regarding the clinical presentation, some characteristics are markedly different in pediatric population. 

First, the tumor volume tends to be larger in patients with less than 20 years old when compared to patients diagnosed between 20 and 50 years [24]. Zimmerman et al. already showed, in 1988 [25], that newly diagnosed tumors were greater than 4 cm in 36% of children as opposed to 15% of adults and had less than 1 cm in 9% of children as opposed to 22% of adults. In series contemplating only patients with papillary carcinoma, only 1.5–3% of tumors had less than 1 cm size at diagnosis [26, 27]. 

Furthermore, probably due to the fact that thyroid volume is smaller in children, an early involvement of thyroid capsule and surrounding tissue is seen [28]. Thus, the category of microcarcinoma (including tumors with less than 1 cm), commonly used in adults, should be avoided in children, since a 1 cm tumor constitutes a very important finding in this age group. 

Secondly, the multicentricity also occurs more frequently in the pediatric age group, especially in the subtype papillary carcinoma [29, 30]. Such outbreaks have been considered as polyclonal in most cases [31]. This becomes especially important as it can be used as an argument in favor of total thyroidectomy as primary surgical approach for these patients. 

Third, pediatric patients have a higher probability of cervical lymph node metastasis as well as distant metastasis [21, 32]. In a series done at the Mayo Clinic with 1039 patients with papillary thyroid carcinoma, cervical lymph node involvement was detected in 90% and metastasis distance in approximately 7% of children versus 35% of cervical lymph node involvement and 2% of distance metastasis in adults [25]. In a study performed by our group with 65 children and adolescents, the occurrence lymph node metastasis at diagnosis was 61.5%, local invasion 39.5%, and distant metastases 29.2%, all of them being in the lungs [33]. As the diagnostic methods improved, clinical presentation of differentiated thyroid carcinoma in the pediatric age group has changed over time. A review held at the University of Michigan comparing patients diagnosed between 1936–1970 with those diagnosed between 1971 and 1990 showed that the patients diagnosed more recently had a lower incidence of lymph node involvement (36% versus 63%), less local invasion (6% versus 31%), and lower incidence of lung metastases (6% versus 19%), reflecting a precocity in diagnosis over the decades, with a consequent better prognosis, particularly if older than 10 years of age [34].

The most common site of distant metastasis in children is the lung with just a few cases described of bone metastases [12, 35] and of central nervous system metastases [12, 36]. 

The histological subtype follows a distribution similar to adults: 90–95% papillary carcinomas and 5% follicular [9, 37, 38]. Poorly differentiated tumors as insular and anaplastic are extremely rare [38].

## 5. Prevalence of Mutations and Expression of NIS

An important difference between thyroid carcinoma in pediatric and adult age is related to the high prevalence of expression of sodium-iodide transporter (NIS) in metastatic focus found in children [39–41]. In the absence of stimulation of TSH, the expression of NIS is undetectable in 65% of papillary tumors and 56% of follicular in patients with less than 20 years [39]. In contrast, the expression of NIS is absent or negligible in 90% of differentiated carcinomas in adults, either when searched by PCR with reverse transcription [40] or by Immunohistochemistry [42]. 

The greater expression of NIS in the pediatric population results in greater responsiveness to radioiodine treatment and better prognosis. In young patients, the recurrence risk increases in those who do not express the protein NIS when compared to those who have it [39]. Thus, the degree of NIS expression correlates with radioiodine avidity by metastases [43] and lower clinical recurrence rates [44]. 

Regarding the molecular biology of these tumors, apparently RET-PTC rearrangements occur in childhood more frequently than in adults, especially in the radiation-related tumors. Initial studies of Chernobyl-associated PTC identified RET/PTC-3 as the most common form of RET rearrangement in radiation-induced childhood PTC [45–49]. However, Pisarchik et al. found that 29% of adult and childhood PTC in Belarus actually contained RET/PTC-1 rearrangements [45]. It was hypothesized that the increase in frequency of the RET/PTC-1 rearrangements in those adults could be related to a longer latency period in those cases. In addition, patients who had RET/PTC-3 rearrangements were diagnosed much earlier after the Chernobyl incident [45]. Motomura et al. reported that 71% of sporadic PTC from children in the United States and 87% of PTC from children living in radiation-contaminated areas of Belarus contain rearrangements of the RET oncogene [50, 51].

Besides RET/PTC rearrangements, other groups suggested the immunohistochemical overexpression of MET associated with high recurrence rate in children and adolescents [51], in addition to the immunohistochemical overexpression of growth factors of vascular endothelium [52] and telomerase, however, without definitive findings [53]. 

In the case of follicular carcinomas, the two most frequently involved genes would be RAS and PPAR gamma, and their rearrangement might serve as a trigger to the transformation from adenoma to carcinoma [54]. However, little is known about its role in the prognosis of such neoplasms.

## 6. Prognosis

The prognosis of these tumors in childhood is a very interesting issue. Despite having a greater recurrence rate when compared to adults, survival seems to be better [55]. Mazzaferri and Kloos in a series with 16.6 years of followup, found a recurrence rate, in patients with less than 20 years old, around 40%, while those with more than 20 years of age had 20% recurrence rates [24]. In contrast, survival is greater than in adults. In a study done in Minsk with a large cohort of 741 patients, the survival rate was 99.3% in 5 years and 98.5% in 10 years in a pediatric population [56]. 

Age seems to be a very important prognostic factor in thyroid cancer. Children and adolescents are usually classified as having a better prognosis and they are classified together with all patients under 45 years old. However, Lazar et al. showed that patients with less than 10 years, mainly prepubertal, had a worse prognosis than the older and more advanced pubertal stages patients [34].

## 7. Treatment

Regardless, the biology of papillary and follicular tumors, the therapeutic approach is very similar for both subtypes of tumors [12, 55]. As well as in adults, the treatment of differentiated thyroid carcinoma is based on the combination of three therapeutic modalities: surgery, hormone replacement with levothyroxine, and radioiodine treatment. The surgery can vary from lobectomy to total thyroidectomy accompanied by cervical lymphadenectomy in various ways. Latest guidelines recommend total thyroidectomy, mainly for larger tumors, 1 cm [24, 57, 58] associated with cervical dissection of central or lateral compartment block if lymph node metastases are seen in preoperative imaging or during the surgery. The main surgical complications include persistent hypoparathyroidism and laryngeal nerve damage that may cause a wide spectrum of clinical consequences: from hoarseness to total vocal cord paralysis, with need for definitive tracheotomy [59]. 

After a total or near-total thyroidectomy, the volume of remaining gland should be less than 2 g seen in the cervical ultrasound performed around one month after surgery [55]. 

Even after total thyroidectomy, some radioiodine uptake is seen in the thyroid bed. Generally, this phenomenon is assigned to the remaining normal thyroid cells left by the surgeon to protect the nerve and around Berry's ligament. However, because multicentricity and metastatic disease are more common in the pediatric age group, the possibility of such outbreaks being malignant cells cannot be ruled out. Thus, most societies recommend radioiodine ablation in the vast majority of patients under 45 years old but none of them make specific recommendations for children and adolescents [55, 58–60]. However, the radioiodine treatment should be used to complement, not replace, the total thyroidectomy. The success of ablation is significantly lower in patients who have undergone less extensive surgery, such as near-total thyroidectomy [24, 61]. In most cases, one dose of radioiodine treatment is capable of achieving complete ablation; however, the procedure may have to be repeated usually 6–12 months after the first [62]. Some variables seem to influence the success of thyroid remnant ablation and the most important one seems to be the presence of lymph node metastases in low risk patients [33]. However, little is known about the prognostic significance of achieving a successful ablation with the first dosage of I-131 in patients with differentiated thyroid cancer. Mazzaferri and Jhiang have shown that adult patients with a successful ablation had a better prognosis than those who failed: disease-free survival was 87% versus 49% after 10 years; additionally, thyroid-cancer related survival was 93% versus 78% [63]. On the other hand, the Mayo Clinic studies did not show a major impact in the overall survival and in the recurrence rates [25, 64].

The third treatment modality is thyroid hormone replacement. This suppressive therapy with thyroid hormone is believed to reduce the risk of growth or tumor proliferation induced by TSH [65]. In children and adolescents still undergoing growth, there are several studies that guarantee the efficacy and safety of this approach, particularly with regard to their final height, as long as they are carefully controlled [55]. 

Possible side effects of long-term suppressive therapy include osteoporosis and cardiovascular disease, especially of left ventricular hypertrophy [65, 66]; such are effects documented in adults.

## 8. Radioiodine in Childhood and Its Side Effects

The radioiodine treatment in pediatric age should be preferably administered in capsule form, in association with an antiemetic medication, in an attempt to ensure that the activity administered has been fully ingested. 

Iodine 131 therapy can lead to a temporary loss of salivary flow and change of taste in up to 30% of the cases [59]. However, permanent xerostomia is rare. The most serious side effect from radioiodine treatment is radiation-induced leukemia that happen in 1 out of 26 treated patients in a study held in Netherlands with children and adolescents [59]. Another concern is pulmonary fibrosis that may occur in up to 1% of cases, mostly in those with diffuse lung metastases. Both effects are dose dependent and usually are seen in patients that underwent multiple treatments with a total dose above 600 mCi [59]. 

The actinic sialoadenitis is common but usually is reversible [67]. This complication is more frequent in the absence of iodine-avid metastases and discrete thyroid remnant, situations with greater availability of radioiodine to the salivary glands [34, 67]. A transitional impairment of spermatogenesis [34, 67, 68] is observed after ablation therapy with high doses of iodine 131. Permanent infertility is possible with accumulated high doses [69]. Usually the production of testosterone is preserved [68, 69], although an elevation of LH can occur [69]. In women, an increment of FSH and reversible menstrual changes [68, 69] and even infertility and early menopause [69] may occur after high doses of radioiodine. 

Whereas the maximum dose absorbed by the gonads is 5 mGy/mCi, Maxon inferred that permanent infertility does not occur in women with doses up to 300 mCi iodine-131 and happen in less than 10% of men with this same dose. With doses of 800 mCi or more, infertility would go up to 60% of women and more than 90% of men [69, 70]. 

In adolescent boys, radioiodine can also cause a decrease in quantity and affect sperm quality leading to infertility that may be transient or permanent [71].

## 9. Controversies

Even with all knowledge acquired today, the controversies on the ideal approach of these patients remain. The lack of studies demonstrating real benefit in overall survival of these patients comparing the different therapeutic modalities contributes to this discussion. Groups like the Mayo Clinic advocate a conservative treatment (considering the possibility of partial thyroidectomy without adjuvant radioiodine therapy) using as argument the observation of 1.7% mortality after 28 years of monitoring and 3.4% recurrence in 30 years in 58 patients under 17 years at diagnosis, in which only 38% underwent total thyroidectomy and 17% radioiodine treatment adjuvant, that is, a good evolution even without the traditionally recommended intensive treatment [25]. 

The main arguments of those who prefer a more aggressive approach are based on studies with long follow-up period analyzing disease-free survival and recurrence rate. For example, Chow et al., in this univariate analysis, showed that the local recurrence rate in children was reduced from 42% to 6.3% when radioiodine adjuvant treatment was performed (*P* = .0001) [72].

The application of the current staging system created by the International Union against Cancer (AJCC/UICC) based on the TNM and age is recommended for all types of tumors including thyroid [73], in an attempt to standardize the tumoral extension description [73]. However, in thyroid carcinoma, TNM staging does not take into consideration several additional factors that influence the evolution and prognosis and so has a limited capacity of predicting outcome in some cases. Thus, several other staging systems are being proposed in the attempt to achieve a better accuracy, among them: CAEORTC, AGES, AMES, MACE, and ATA. [58, 74–77]. These systems take into account factors identified as predictor of outcomes in retrospective studies, usually taking into consideration the presence of metastases, the age of the patient, and the extent of the tumor site. However, most of them were developed to predict cancer-specific mortality not to predict recurrence [76]. Because the mortality is low, there is not an ideal standing system for thyroid cancer yet, especially when it comes to the pediatric population. These patients are usually grouped with minors 45 years which may be responsible for the low accuracy of all existing systems for patients under 20 years of age, that clearly have a different clinical presentations and biology when compared to older patients. In a recent study performed with 65 patients under 20 years old, the staging system proposed by ATA in 2009 seems to be better than the others for predicting disease-free survival [33]. More studies with this specific population are needed to develop a specific risk assessment for this age group.

## 10. Conclusion

Although children with DTC typically present with locoregional metastases and a high rate of distant metastatic disease, overall survival is very good. Treatment should be based on their increased risk for recurrence instead of overall mortality, and lifelong followup is required because recurrence and death may not occur for decades after diagnosis. Initial treatment will generally include total thyroidectomy and central compartment lymph node dissection especially if lymph node disease is found in the preoperative evaluation. Radioiodine ablation should be individualized and given to those with a higher risk of recurrence.

Large multicenter studies are needed to better understand optimal treatment approaches to this unique population. All care of pediatric DTC should be delivered by multidisciplinary specialized teams which include both pediatricians and thyroid cancer specialists to minimize possible complications and ensure competent followup.
